# Travel-Related Venous Thrombosis: Results from a Large Population-Based Case Control Study (MEGA Study)

**DOI:** 10.1371/journal.pmed.0030307

**Published:** 2006-08-22

**Authors:** Suzanne C Cannegieter, Carine J. M Doggen, Hans C van Houwelingen, Frits R Rosendaal

**Affiliations:** 1 Department of Clinical Epidemiology, Leiden University Medical Center, Leiden, Netherlands; 2 Department of Medical Statistics, Leiden University Medical Center, Leiden, Netherlands; 3 Thrombosis and Haemostasis Research Center, Department of Haematology, Leiden University Medical Center, Leiden, Netherlands; McGill University, Canada

## Abstract

**Background:**

Recent studies have indicated an increased risk of venous thrombosis after air travel. Nevertheless, questions on the magnitude of risk, the underlying mechanism, and modifying factors remain unanswered.

**Methods and Findings:**

We studied the effect of various modes and duration of travel on the risk of venous thrombosis in a large ongoing case-control study on risk factors for venous thrombosis in an unselected population (MEGA study). We also assessed the combined effect of travel and prothrombotic mutations, body mass index, height, and oral contraceptive use.

Since March 1999, consecutive patients younger than 70 y with a first venous thrombosis have been invited to participate in the study, with their partners serving as matched control individuals. Information has been collected on acquired and genetic risk factors for venous thrombosis. Of 1,906 patients, 233 had traveled for more than 4 h in the 8 wk preceding the event. Traveling in general was found to increase the risk of venous thrombosis 2-fold (odds ratio [OR] 2.1; 95% confidence interval [CI] 1.5–3.0). The risk of flying was similar to the risks of traveling by car, bus, or train. The risk was highest in the first week after traveling. Travel by car, bus, or train led to a high relative risk of thrombosis in individuals with factor V Leiden (OR 8.1; 95% CI 2.7–24.7), in those who had a body mass index of more than 30 kg/m^2^ (OR 9.9; 95% CI 3.6–27.6), in those who were more than 1.90 m tall (OR 4.7; 95% CI 1.4–15.4), and in those who used oral contraceptives (estimated OR > 20). For air travel these synergistic findings were more apparent, while people shorter than 1.60 m had an increased risk of thrombosis after air travel (OR 4.9; 95% CI 0.9–25.6) as well.

**Conclusions:**

The risk of venous thrombosis after travel is moderately increased for all modes of travel. Subgroups exist in which the risk is highly increased.

## Abstract 2

In a large case-control study of travelers, the risk of venous thrombosis was increased 3-fold with a similar risk for flying or travelling by car, bus, or train.

## Editors' Summary

**Background.**

Recently there has been increasing concern that blood clots (thromboses) in the leg or lungs occur with greater frequency after air travel. Several theories have been put forward to explain why this increase might happen, including the fact that air passengers tend to not move around much, or possibly that reduced amounts of oxygen in the blood make the blood more likely to clot. Understanding what causes such clots is important as it would help us come up with suggestions of ways to prevent them.

**Why Was This Study Done?**

It is not possible to test in a controlled trial whether travel causes an increase in blood clots, so the next best way of studying this problem is to do a case-control study, in which people with blood clots (cases) are compared with similar people who don't have a blood clot (controls—in this case, the partners of the cases), and the differences in a number of contributing factors are assessed.

**What Did the Researchers Do and Find?**

Since 1999, the MEGA (Multiple Environmental and Genetic Assessment of Risk Factors for Venous Thrombosis) study has aimed to identify all people in an area of the Netherlands who develop a blood clot for the first time, by seeking out people who receive treatment for blood clots. At the time of this report, 1,906 people with clots had been found; of these, 233 had traveled for more than four hours in the eight weeks preceding the event. Traveling in general was found to increase the risk of clots two-fold, and the risk was highest in the week after traveling. The risk of flying was similar to the risk of traveling by car, bus, or train, and was highest in the first week after traveling. Certain other factors increased the risk of a blood clot even more, such as having a particular mutation (known as factor V Leiden) in a gene involved in blood clotting, having a body mass index of more than 30 kg/m^2^ (over 30 kg/m^2^ is defined as being obese), being more than 1.90 meters tall, and using oral contraceptives. All these factors made the risk of clots especially after air travel worse; in addition, people shorter than 1.60 meters also had an increased risk of thrombosis after air travel. However, it should be borne in mind that the number of cases in each of these various groups was quite small, and the overall risk of getting a thrombosis is still low.

**What Do These Findings Mean?**

Since the risks of thrombosis are increased for all types of long travel, it seems that the main factor causing the thrombosis is immobility. However, since the risk is even higher for air travel, the relative lack of oxygen may also play a part. One interesting aspect of this study is that the researchers used partners as controls; in order to be sure that doing this did not make the results invalid, the researchers had to carefully adjust for differences between the cases and controls, such as the fact that partners were generally of the opposite sex. In a related Perspective (DOI: 10.1371/journal.pmed.0030300), Kenneth Rothman discusses the study further.

**Additional Information.**

Please access these Web sites via the online version of this summary at http://dx.doi.org/10.1371/journal.pmed.0030307.

- MedlinePlus encyclopedia entries on deep venous thrombosis and pulmonary embolus
- OMNI, a health information service in the UK run by the Resource Discovery Network, has links to pages of information on venous thrombosis
- The Web site for the MEGA study in this paper gives further background and information
- The Web site of the World Health Organization Research Initiative into Global Hazards of Travel has information on research into the connection between air travel and venous thrombosis

## Introduction

Interest in the role of air travel in the pathogenesis of venous thrombosis has heightened in the past 5 y [1–5]. Venous thrombosis was first linked to air travel in 1954 [6], and as air travel has become more and more common, many case reports and case series have been published since. Several clinical studies have shown an association between air travel and the risk of venous thrombosis. In a series of individuals who died suddenly at Heathrow Airport, death occurred far more often in the arrival than in the departure area [7]. Two similar studies described a “dose-response” relation: the risk of pulmonary embolism in air travelers increased with the distance traveled [5,8]. A number of case-control studies, however, have shown conflicting results [9–11]. More recently, a 2-fold increased risk in patients who had traveled by air was described in a case-control study among 210 patients and 210 controls [3]. A case-crossover study based on record linking in Australia described a 4-fold increased risk of venous thrombosis in the first 2 wk after a long-haul flight [1]. In terms of absolute risk, two studies found similar results: one performed in New Zealand found a frequency of 1% of venous thrombosis in 878 individuals who had traveled by air for at least 10 h [2], and a German study found venous thrombotic events in 2.8% of 964 individuals who had traveled for more than 8 h in an airplane, as compared to 1% in 1,213 controls [4]. The events in both studies were mostly asymptomatic.

The available evidence suggests that the overall risk of venous thrombosis is moderately increased after air travel. Nevertheless, many questions remain unanswered: the exact underlying mechanism is still unknown, and, related to this, it is not clear whether the risk is increased after air travel only or after long-distance travel in general. Furthermore, the effect of the combination of other risk factors for venous thrombosis and travel has not yet been systematically studied, with the exception of a study by Martinelli et al., who found an additionally increased risk in patients with thrombophilia and patients who used oral contraceptives [3].

The Multiple Environmental and Genetic Assessment (MEGA) study of risk factors for venous thrombosis is a large ongoing case-control study aimed at assessing the combined effect of genetic and acquired risk factors for venous thrombosis. Cases and control individuals are questioned about—among many other items—travel that occurred shortly before the event. This provides an opportunity to assess the effect of travel on the risk of thrombosis in an unselected population, as well as the effect of the combination of travel with several other risk factors for thrombosis.

## Methods

### Study Design

Since March 1999, consecutive patients younger than 70 y with a first deep-vein thrombosis (DVT) or pulmonary embolism (PE) have been identified at six regional anticoagulation clinics in the Netherlands. Anticoagulant clinics monitor the anticoagulant therapy of all patients in a well-defined geographical area, allowing us to identify consecutive and unselected patients with thrombosis. Patients who were unable to fill in the questionnaire (because of language or severe psychiatric problems), as well as those who died soon after the venous thrombosis or who were in the end stage of a disease and for that reason did not participate, were not included. All others were considered eligible. Partners of these patients were invited as control individuals, and the same exclusion criteria were applied.

All participants filled in a detailed standardized questionnaire on general demographic and anthropomorphic characteristics, as well as risk factors for venous thrombosis. The questionnaire was sent to all participants within a few weeks after the event and covered the period of 1 y prior to the date of the thrombotic event (index date). When the participant was unable to fill in the questionnaire we asked questions by phone, using a standardized mini-questionnaire. Three months after the patients had discontinued their oral anticoagulant therapy, they were invited with their partners to the anticoagulation clinic for a blood sample. In those patients who continued to take oral anticoagulant therapy for more than 1 y after the event, blood was drawn during therapy. If participants were unable to come to the clinic, a buccal swab was sent by mail to replace the blood sample for DNA extraction.

The study protocol was approved by the Ethics Committee of the Leiden University Medical Center. Written informed consent was obtained from all participants [12].

### Validation Study of Thrombosis Diagnosis

Discharge letters or diagnostic reports of the venous thrombotic event were obtained for a sample of 742 patients who had their first thrombosis between March 1999 and March 2000. The diagnostic management of the patients was compared to the diagnostic procedure as described in the Dutch consensus [13]. Diagnosis of clinically suspected DVT of the leg is based on a clinical score, serial compression ultrasonography, and D-dimer assay. Objective testing of clinically suspected pulmonary embolism is based on perfusion and ventilation scintigraphy, ultrasonography of the leg veins, pulmonary angiography, or helical computed tomography. Out of 395 patients with DVT of the leg, 384 (97%) were objectively diagnosed, while out of 347 patients with PE, 271 (78%) were confirmed with objective testing as certainly having PE. Since the diagnosis appears to be made by objective methods in virtually all cases of DVT, while being more ambiguous for PE, we also analyzed these two manifestations of venous thrombosis separately.

### Current Analysis

For the current analysis we were interested in the effects of travel, and its combined effect with other common risk factors for venous thrombosis. Patients with a solitary arm thrombosis were excluded from this analysis. Of 3,902 eligible cases, diagnosed up to May 2002, 656 did not participate for various reasons (such as not willing or not reachable), leading to a response of 83%. A further 3% responded only to the mini-questionnaire, taken by phone, which did not contain questions about travel. Of the remaining 3,111 cases, 78% had a partner, 77% of whom were willing to participate, which left 1,867 couples. Additionally, 229 partners were identified for whom the corresponding patient originally participated but was later found not to be eligible (aged over 70 y, or not a first thrombotic event). These control individuals were matched on sex and 5-y age groups to one of the 557 patients whose partner did not want to participate, so an extra 229 pairs were included, making a total of 4,192 participants (2,096 pairs). As part of the general questionnaire, questions had been asked about whether or not respondents had traveled for more than 4 h in the 3 mo before the index date, about the travel date, and about mode and duration of travel. We assessed the occurrence of thrombosis in relation to the period of time that had passed since traveling. Travel was defined in the analysis as at least one journey with a duration of at least four uninterrupted hours during the 8-wk period before the event. During the analysis it appeared that some individuals had provided dates of travel *after* the event instead of before. As there was only one opportunity to fill in such a date, we had no information about the period before the event. This was the case in 88 cases and 146 controls. We excluded these individuals and their partners, which left 3,812 participants (1,906 pairs) for the analysis.

Because we selected the partners of the cases as control individuals, and because it turned out, as expected, that couples tend to travel together, we performed a conditional logistic regression analysis to calculate odds ratios (ORs) for the relation between travel and venous thrombosis. This method fully takes this matching into account, and leads to unbiased estimates, with adjustment for all factors in which cases and controls tend to be similar, e.g., socioeconomic class [14]. Details of this method can be found in Protocol S1. The 95% confidence intervals (CIs) were derived from the model.

We assessed the combined effect of traveling and the following risk factors for thrombosis: factor V Leiden mutation, prothrombin G20210A mutation, body mass index (BMI, as kg/m^2^), and height. We were also interested in the combined effect of oral contraceptive use and travel. However, as the control individuals were nearly always of the opposite sex (partners of the cases were recruited as controls), it was not possible to perform a matched analysis for the combination of oral contraceptive use and travel. Therefore, we performed a case-only analysis [15]. This method allows one to examine the association between two exposures among case individuals only. ORs are interpreted as a synergy index (SI) on a multiplicative scale, with independence assumed between the exposures. As this analysis depends only on cases, it was possible to perform it in all consecutive cases, therefore also including those without a partner.

### Laboratory Measurements

Blood was collected from the antecubital vein into vacuum tubes containing 0.106 mol/l trisodium citrate. High molecular weight DNA was isolated from leukocytes using a standard salting-out procedure [16] and stored at −20 °C. When a blood sample was not available, DNA was extracted from buccal swabs. Three large cotton swabs in a total of 6 ml of SDS–proteinase K solution (100 mM NaCl, 10 mM EDTA, 10 mM Tris-HCl [pH 8.0], 0.5% SDS, 0.1 mg/ml proteinase K) were obtained. Upon arrival, the proteinase K concentration was raised to 0.2 mg/ml, and the sample was incubated for 2 h at 65 °C. Subsequently, the solute was recovered by centrifugation. Potassium acetate was added to the supernatant to a final concentration of 1.6 M. After 15 min incubation on ice, proteins were removed using chloroform/isomylalcohol (24:1) treatment. The DNA in the water phase was subsequently ethanol precipitated. After centrifugation, the pellet was resuspended in 200 μl of 10 mM Tris-HCl and 10 mM EDTA (pH 8.0), and frozen at −20 °C until further analysis. The factor V Leiden mutation (G1691A) and the prothrombin mutation (G20210A) were simultaneously detected by duplex polymerase chain reaction [17,18]. The technician was blinded concerning the origin of the sample, i.e., whether it was from a patient or from a control individual.

## Results

### Venous Thrombosis in Relation to Travel

Table 1 shows general characteristics of the 1,906 patients. They ranged in age from 18 to 69 y (median 50.4 y); 51% were men. Diagnosis was DVT in 57% of the cases, PE in 32%, and both in 11%. As partners of the cases were included as control individuals, the sex distribution of the control individuals was the opposite; the age distribution differed only trivially.

**Table 1:**
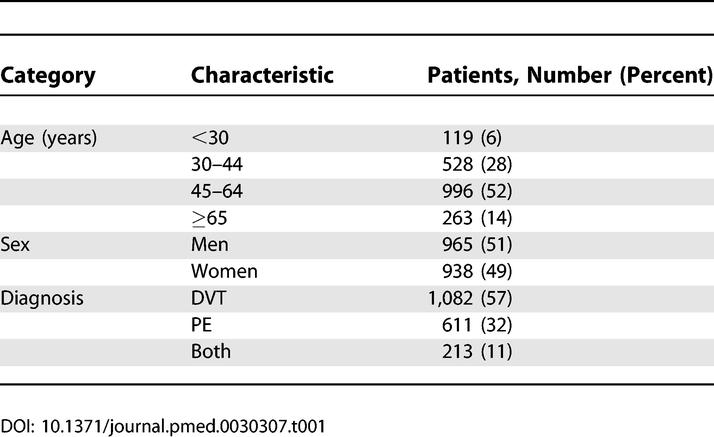
Characteristics of the 1,906 Patients with Venous Thrombosis

Of the patients, 233 individuals (12%) had traveled for at least 4 h by air, bus, car, or train within the 8 wk preceding the index date, as compared to 182 of the control individuals (9.5%). As the cases and control individuals were selected as couples, many pairs (135) had traveled together and were uninformative: as a consequence, 145 pairs in which either the patient (98) or the control (47) had traveled could be used for the matched analysis (Table 2). This analysis showed a 2-fold increased risk of venous thrombosis for all modes of travel combined (OR 2.1; 95% CI 1.5–3.0) compared to not traveling. For air travel alone, 49 individuals (31 cases and 18 controls) had traveled without their partner, and the analysis yielded an OR of 1.7 (95% CI 1.0–3.1). For the other modes of travel (car, bus, and train) the relative risks were essentially similar to each other and to that of air travel (Table 2).

**Table 2:**
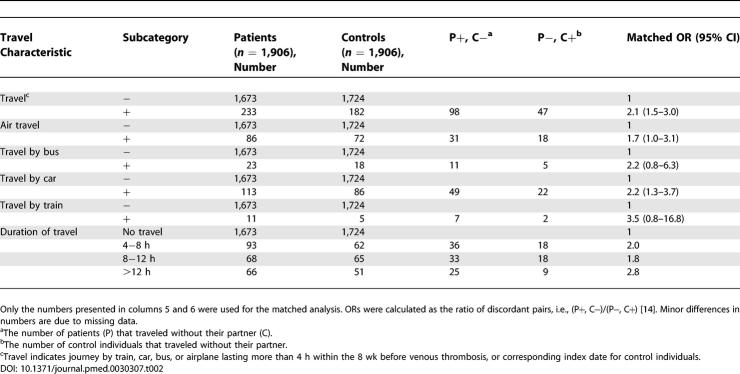
ORs for Venous Thrombosis in Relation to Travel

**Figure 1:**
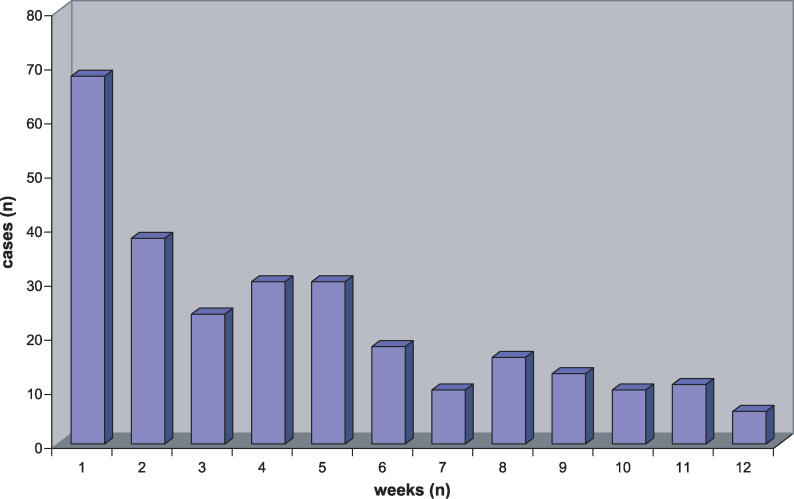
Frequency of the Occurrence of Events within the First 12 wk after Travel The time window of analysis concerned the first 8 wk.

The risk of venous thrombosis was not clearly related to increased duration of travel (Table 2). Of the 233 events that occurred within 8 wk after traveling, 68 (29%) were diagnosed in the first week, after which the incidence gradually decreased (Figure 1).

### The Effect of Other Risk Factors Combined with Travel

#### Prothrombotic mutations.

Information on the factor V Leiden mutation and prothrombin G20210A genotype was available for 1,713 patients (90%) and for 1,629 of the control individuals (85%). Factor V Leiden was present in 259 cases (14%) and 84 control individuals (4%) (OR 3.1; 95% CI 2.4–4.1).

The risk of venous thrombosis was 8-fold increased in people with factor V Leiden who had traveled by bus, car, or train (modes combined) as compared to noncarriers who did not travel (OR 8.1; 95% CI 2.7–24.7). For the combined effect of air travel and factor V Leiden, the risk seemed even slightly higher (OR 13.6; 95% CI 2.9–64.2).

The prothrombin G20210A mutation was found in 83 cases (4%) and in 29 control individuals (2%) (OR 2.7; 95% CI 1.7–4.2). The risk in individuals with this mutation who had traveled was difficult to interpret because of the small numbers but appeared not to increase more than additively (Table 3).

**Table 3:**
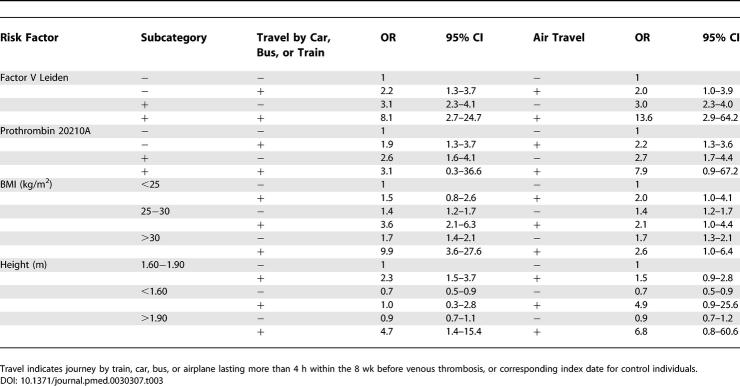
The Combined Effect of Other Risk Factors and Travel on the Risk of Venous Thrombosis (DVT, PE, and Both; 1,906 Pairs)

#### BMI.

The effect of BMI was studied by dividing individuals into three categories with the following BMI values: <25, 25–30, and >30 kg/m^2^ [19]. A BMI of 25–30 kg/m^2^ was associated with an increased risk of venous thrombosis (OR 1.4; 95% CI 1.2–1.7), and the risk was slightly higher in patients with a BMI of 30 kg/m^2^ or more (OR 1.7; 95% CI 1.4–2.1).

The combined effect of a higher BMI and travel was the sum of the individual risks (Table 3), with the exception of people with a BMI of more than 30 kg/m^2^ who traveled by car, bus, or train, for whom the risk was 10-fold increased (OR 9.9; 95% CI 3.6–27.6). This increase in risk was not found in people who traveled by air.

#### Height.

Particularly short or tall people may be subjected during travel to even more unnatural sitting positions than individuals with average height. Therefore, we assessed the effect of extremes of heights in combination with travel on the risk of venous thrombosis by comparing short (less than 1.60 m) and tall individuals (more than 1.90 m) with people of average height (1.60–1.90 m). Compared to people of average height, the risk of venous thrombosis was lower for short people (OR 0.7; 95% CI 0.5–0.9) and did not differ for very tall individuals (OR 0.9; 95% CI 0.7–1.1). The risk was found to be increased in people of more than 1.90 m who traveled (OR 4.7; 95% CI 1.4–15.4 for travel by car, bus, or train; OR 6.8; 95% CI 0.8–60.6 for air travel) compared to non-traveling people of average height. Interestingly, the risk of venous thrombosis was also increased in short people but only after air travel (OR 4.9; 95% CI 0.9–25.6), not after other modes of travel (OR 1.0; 95% CI 0.3–2.8, all relative to non-traveling people of average height).

#### Oral contraception.

To study the association between oral contraceptive use, travel, and the risk of venous thrombosis, we performed a case-only analysis in all female patients who were less than 50 y of age. As we needed only cases, it was also possible to include women without a partner for this analysis, which led to a total of 1,025 women aged under 50. Non-users who did not travel were used as the reference group. The case-only estimate of the SI for women who traveled by car, bus, or train was 2.4 (95% CI 1.5–3.7). This indicates that the OR for the combination of travel and oral contraceptive use is 2.4 times the product of the separate ORs. As oral contraceptive use generally increases the risk of venous thrombosis about 4-fold [20], the combination with travel by car, bus, or train would lead to an estimated OR of about 20 (4 × 2 × 2.4). A clearly stronger interaction of travel by air with oral contraceptive use was found: the case-only estimate of the SI was 4.9 (95% CI 2.1– 11.4), which would result in an OR of about 40 (4 × 2 × 4.9).

### Effect of Risk Factors in DVT Patients Only

Of the 1,906 cases, 1,082 were diagnosed with DVT. As the diagnosis was more unambiguous in these patients (97% objectively diagnosed as compared to 78% of the PE patients), we repeated the analysis in these patients only.

In this analysis, the overall effect of travel on the risk of DVT was equal to the effect on all venous thrombosis (DVT and PE combined). However, here we found a stronger risk for travel by air (OR 3.0; 95% CI 1.3–7.1) then for travel by car, bus, or train (OR 1.9; 95% CI 1.1–3.2) (Table 4). Also, the analysis of the combination of other risk factors with travel resulted in more clear-cut effects, despite the smaller number of cases: the risk of DVT was still clearly synergistically increased in patients with factor V Leiden who traveled, whereas the prothrombin G20210A mutation did not further increase the risk of travel (Table 4). Furthermore, a BMI of more than 30 kg/m^2^ in combination with travel yielded high ORs for DVT both in people who traveled by car, bus, or train and in those who flew. Being more than 1.90 m tall in combination with travel resulted in higher ORs for DVT; the risk for short people was more increased after travel by air (OR 6.8; 95% CI 1.1–43.5) (Table 4). The effect of oral contraceptive use in combination with travel by car, bus, or train on the risk of DVT was studied in 589 women and was somewhat lower than the effect on the risk of all venous thrombosis (SI 1.9; 95% CI 0.9–4.2). In those who traveled by air it was also a bit lower (SI 3.4; 95% CI 1.3–8.8), but still indicative of a strong synergistic effect.

**Table 4:**
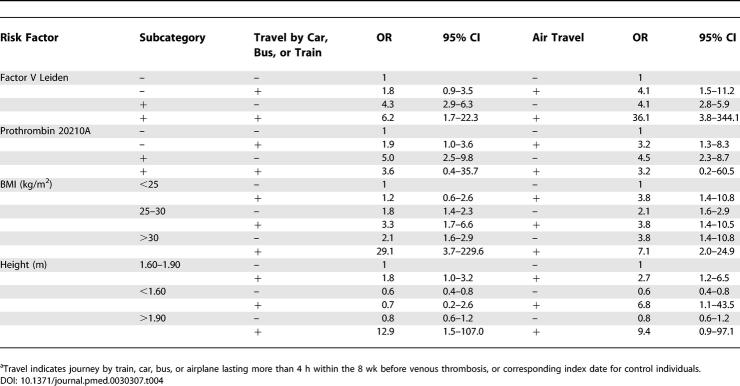
The Combined Effect of Other Risk Factors and Travel on the Risk of DVT Only (1,082 Pairs)

## Discussion

In this population-based case-control study, long-distance traveling increased the risk of venous thrombosis 2-fold. Travel by air increased the risk to the same extent as travel by car, bus, or train. The risk was highest in the first week after traveling. As venous thrombosis is a disease in which many factors (genetic and acquired) interact [21], we identified groups with additional risk factors in which the risk was further increased. This was the case for individuals with factor V Leiden, obese people (BMI > 30 kg/m^2^), and short (only for travel by air) and tall people, as well as for women using oral contraceptives. Some of these synergistic effects were more apparent for air travel.

Although the studies that have been published so far have not yielded entirely consistent results, those that did report an increased risk of venous thrombosis in air travelers showed similar risk estimates of a 2- to 3-fold increased risk (even in one with asymptomatic events only [4]). The occurrence of venous thrombosis was highest in the first week after travel, and slowly declined afterwards, a pattern that was also described in a recent record-linking study from Australia [1], supporting a causal relation.

As a possible mechanism for an extra risk in travelers who fly, an effect of hypobaric hypoxia on the coagulation system was postulated, which has already been studied a number of times, mainly in hypobaric chambers, with unclear results so far. Our study showed an increased risk in all types of travel, which suggests that the increased risk of flying is caused mainly by immobilization. Additionally, the risk is further increased in short and tall people, who are likely to experience more immobilization and venous compression than other travelers. However, as some of our findings were more pronounced for air travel, we cannot exclude an additional effect of hypobaric hypoxia, possibly in risk groups only. This possibility is supported by a recent study of our group [22] in which we found that thrombin generation occurred in some healthy volunteers after flying for 8 h but happened to a far lesser extent after being immobilized for 8 h in a cinema. The high response in the fliers was associated with the presence of risk factors for thrombosis, i.e., oral contraceptive use, the factor V Leiden mutation, and the combination of the two. This finding indicates an effect of an additional factor in an airplane, such as hypobaric hypoxia, to which mainly individuals with risk factors respond.

None of the studies published so far have systematically studied the effect of traveling in combination with other risk factors, with the exception of the study by Martinelli et al. [3]. In an analysis of 210 patients, they found a 16-fold increased risk for patients who traveled by air and had some form of thrombophilia, as well as a 14-fold increased risk in women who flew and used oral contraceptives, findings that confirm both the results of the present study and our finding of activated coagulation in individuals with risk factors after flying [22].

The finding that taller and shorter people had an increased risk of venous thrombosis after traveling should be interpreted with some caution, as the numbers were small in these strata. On the other hand, it is biologically plausible: very tall people are subjected to even more cramped seating than average-height individuals, and very short people's feet may not touch the floor, which would lead to extra compression of the popliteal veins. Interestingly, the increased risk for short people was only found in people who traveled by air. This may have to do with the fact that seats in cars are generally lower, and more individually adjustable, than those in airplanes.

As the diagnosis of DVT is usually more unambiguous than that of PE [23], as was the case in our study population as well, we repeated the analysis using only DVT as the outcome of interest (97% objectively diagnosed). In this analysis, despite using smaller numbers, most findings were either similar or appeared more evident, and inconsistencies that were found when using both DVT and PE as endpoints disappeared.

To our knowledge, this is the first large population-based case-control study in which the effect of travel on the risk of venous thrombosis has been studied. Because the control individuals were closely matched, being partners of the cases, and couples tend to travel together, only the cases and control individuals who had not traveled together could be used for the analysis. Also because of this design, the effect of sex and age could not be studied. It has to be noted, however, that for all other research questions on the effect of genetic and acquired risk factors on the risk of venous thrombosis, this design has no limitations and the close matching of cases and controls renders confounding by, for instance, lifestyle and socioeconomic class less likely than in previous unmatched studies (see also Protocol S1). Another advantage of this approach is the minimization of recall bias, as the cases and controls would generally fill in the questionnaire together.

Many questions are still left unanswered that necessitate more research. First of all, our study results apply only to people younger than 70 y of age. Furthermore, it is likely that other characteristics exist that also increase the risk—person-specific (e.g., other drug use), behavioral (e.g., use of sleeping pills or alcohol consumption), and flight-specific (e.g., class or seating)—that need to be identified. These further variables are part of our ongoing study as part of the World Health Organization Research Initiative into the Global Hazards of Travel (WRIGHT study). For those who have an increased risk, such as oral contraceptive users and individuals with factor V Leiden, prevention may be warranted. Prevention may vary from simple measures, such as exercises during the flight, to measures that carry a risk themselves, such as anticoagulants. Specific studies are needed to assess the efficacy of these measures and their risk–benefit ratio.

It can be concluded that the risk of venous thrombosis is 2-fold increased for all travelers and to the same extent for all modes of travel. In individuals who use oral contraceptives, are carriers of the factor V Leiden mutation, or are particularly tall, short, or obese, this risk is considerably higher, to such an extent that studies into the efficacy of prophylactic measures are required.

## Supporting Information

Alternative Language Abstract S1 Translation of the Abstract into Dutch by Author (25 KB DOC)

Protocol S1 Methodological Appendix (210 KB DOC)

## Abbreviations

BMI: body mass index
CI: confidence interval
DVT: deep-vein thrombosis
OR: odds ratio
PE: pulmonary embolism
SI: synergy index

## References

[pmed-0030307-b001] Kelman CW, Kortt MA, Becker NG, Li Z, Mathews JD (2003). Deep vein thrombosis and air travel: Record linkage study. BMJ.

[pmed-0030307-b002] Hughes RJ, Hopkins RJ, Hill S, Weatherall M, Van de Water N (2003). Frequency of venous thromboembolism in low to moderate risk long distance air travellers: The New Zealand Air Traveller's Thrombosis (NZATT) study. Lancet.

[pmed-0030307-b003] Martinelli I, Taioli E, Battaglioli T, Podda GM, Passamonti SM (2003). Risk of venous thromboembolism after air travel: Interaction with thrombophilia and oral contraceptives. Arch Intern Med.

[pmed-0030307-b004] Schwarz T, Siegert G, Oettler W, Halbritter K, Beyer J (2003). Venous thrombosis after long-haul flights. Arch Intern Med.

[pmed-0030307-b005] Perez-Rodriguez E, Jimenez D, Diaz G, Perez-Walton I, Luque M (2003). Incidence of air travel-related pulmonary embolism at the Madrid-Barajas airport. Arch Intern Med.

[pmed-0030307-b006] Homans J (1954). Thrombosis of the leg veins due to prolonged sitting. N Engl J Med.

[pmed-0030307-b007] Sarvesvaran R (1986). Sudden natural deaths associated with commercial air travel. Med Sci Law.

[pmed-0030307-b008] Lapostolle F, Surget V, Borron SW, Desmaizieres M, Sordelet D (2001). Severe pulmonary embolism associated with air travel. N Engl J Med.

[pmed-0030307-b009] Ferrari E, Chevallier T, Chapelier A, Baudouy M (1999). Travel as a risk factor for venous thromboembolic disease: A case-control study. Chest.

[pmed-0030307-b010] Kraaijenhagen RA, Haverkamp D, Koopman MM, Prandoni P, Piovella F (2000). Travel and risk of venous thrombosis. Lancet.

[pmed-0030307-b011] Arya R, Barnes JA, Hossain U, Patel RK, Cohen AT (2002). Long-haul flights and deep vein thrombosis: A significant risk only when additional factors are also present. Br J Haematol.

[pmed-0030307-b012] Blom JW, Doggen CJM, Osanto S, Rosendaal FR (2005). Malignancies, prothrombotic mutations and the risk of venous thrombosis: Results of the MEGA study. JAMA.

[pmed-0030307-b013] Büller HR, van der Meer J, Oudkerk M (2000). CBO-richtlijn ‘Diep veneuze trombose en longembolie'; herziening van eerdere richtlijnen. Ned Tijdschr Geneeskd.

[pmed-0030307-b014] Breslow NE, Day NE (1980). The analysis of case-control studies. Volume 1, Statistical methods in cancer research.

[pmed-0030307-b015] Khoury MJ, Flanders WD (1996). Non-traditional epidemiologic approaches in the analysis of gene-environment interaction: Case-control studies with no controls!. Am J Epidemiol.

[pmed-0030307-b016] Miller SA, Dykes DD, Polesky HF (1988). A simple salting out procedure for extracting DNA from human nucleated cells. Nucleic Acids Res.

[pmed-0030307-b017] Bertina RM, Koeleman BP, Koster T, Rosendaal FR, Dirven RJ (1994). Mutation in blood coagulation factor V associated with resistance to activated protein C. Nature.

[pmed-0030307-b018] Poort SR, Rosendaal FR, Reitsma PH, Bertina RM (1996). A common genetic variation in the 3′-untranslated region of the prothrombin gene is associated with elevated plasma prothrombin levels and an increase in venous thrombosis. Blood.

[pmed-0030307-b019] Abdollahi M, Cushman M, Rosendaal FR (2003). Obesity: Risk of venous thrombosis and the interaction with coagulation factor levels and oral contraceptive use. Thromb Haemost.

[pmed-0030307-b020] Rosendaal FR, van Hylckama Vlieg A, Tanis BC, Helmerhorst FM (2003). Estrogens, progestogens and thrombosis. J Thromb Haemos.

[pmed-0030307-b021] Rosendaal FR (1999). Venous thrombosis: A multicausal disease. Lancet.

[pmed-0030307-b022] Schreijer AJM, Cannegieter SC, Meijers JCM, Middeldorp S, Büller HR (2006). Activation of coagulation system during air travel: A crossover study. Lancet.

[pmed-0030307-b023] The PIOPED Investigators (1990). Value of the ventilation/perfusion scan in acute pulmonary embolism. Results of the prospective investigation of pulmonary embolism diagnosis (PIOPED). The PIOPED Investigators. JAMA.

