# Supplementary material for: Travel-Related Venous Thrombosis: Results from a Large Population-Based Case Control Study (MEGA Study)
Source: PLoS Med. 2006 Aug 22;3(8):e307. doi: 10.1371/journal.pmed.0030307 (PMC1551914; doi:10.1371/journal.pmed.0030307)
Supplement: Supplementary file 1 [file pmed.0030307.sd001.doc]

**Samenvatting**

**Achtergrond**

Recent onderzoek heeft uitgewezen dat langdurig reizen per vliegtuig een verhoogd risico op veneuze trombose met zich meebrengt. De grootte van het risico is niet precies bekend, noch welke factoren het risico beïnvloeden, noch wat het onderliggende mechanisme is. In een groot patiënt-controle onderzoek naar risicofactoren voor veneuze trombose (MEGA-studie) hebben wij het effect van verschillende manieren van reizen en de reisduur op het ontstaan van veneuze trombose onderzocht. Hierbij keken we ook wat het risico was in combinatie met andere risicofactoren voor trombose, zoals trombofilie, Quételet index, lengte en gebruik van orale anticonceptie .

**Methoden en resultaten**

Opeenvolgende patiënten met een eerste veneuze trombose, jonger dan 70 jaar, werden sinds maart 1999 uitgenodigd voor de MEGA-studie, waarbij hun partners gevraagd werden als controlepersoon. Van alle deelnemers werd informatie verkregen over genetische en verworven risicofactoren voor trombose. Van de 1906 patiënten hadden er 233 langer dan 4 uur gereisd in een periode van 8 weken voor de trombose. Hieruit werd berekend dat in de acht weken na een lange reis het risico op trombose twee maal verhoogd was(odds ratio (OR) 2.1; 95% betrouwbaarheidsinterval (95% CI) 1.5-3.0). Het risico op trombose was verhoogd zowel bij reizen per vliegtuig als bij reizen per auto, trein of bus. Het risico op trombose was het hoogst in de eerste week na het reizen. Reizen per auto, trein of bus leidde tot een hoog relatief risico op trombose in mensen met factor V Leiden (OR 8.1; 95% CI 2.7-24.7), in mensen met een Quételetindex (QI) van meer dan 30 kg/m2 (OR 9.9; 95% CI 3.6-27.6), in mensen met een lengte van meer dan 1.90 m (OR 4.7; 95% CI 1.4-15.4), en in pilgebruiksters (geschatte OR >20). Voor reizen per vliegtuig waren deze bevindingen zelfs nog meer uitgesproken en bleken bovendien mensen met een lengte van minder dan 1.60 m ook een verhoogde kans op het krijgen van trombose te hebben (OR 4.9; (95% CI 0.9-25.6), t.o.v. een lichaamslengte tussen 1.60 en 1.90m).

**Conclusie**

Het risico op trombose is verhoogd na reizen van meer dan vier uur, en dit is het geval voor ieder vervoermiddel. Er zijn subgroepen met een sterk verhoogd relatief risico op trombose na reizen.
