# Supplementary material for: Travel-Related Venous Thrombosis: Results from a Large Population-Based Case Control Study (MEGA Study)
Source: PLoS Med. 2006 Aug 22;3(8):e307. doi: 10.1371/journal.pmed.0030307 (PMC1551914; doi:10.1371/journal.pmed.0030307)
Supplement: Supplementary file 2 [file pmed.0030307.sd002.doc]

**Methodological appendix**

**Matching: general considerations**

Confounding is an issue that arises in almost every epidemiological study. Briefly, this occurs when other factors (that are not the interest of the study) are present that influence the association of interest between exposure and outcome. There are several ways to deal with the problem of confounding, one of which is matching. As matching is a complicated and counterintuitive method, it is generally used only when its advantages outweigh its disadvantages. Matching can be useful when it increases the efficiency of a study and when confounding factors are unknown or difficult to measure.

When matching is done according to a strong confounder, it tends to increase the statistical power of the study. It can also be more efficient to match when a strong confounder will produce such an imbalance between cases and controls that adjustment for this factor is difficult or even impossible. For example, in a case-control study of risk factors for prostate cancer, the cases will come from the older age groups. When controls from all age groups are assembled, there may not be enough overlap with the age range of the cases, especially when the sample size is small.

For most of the methods that deal with confounding, the possible confounding factors need to be known and identified. Factors that are difficult to measure or are unknown (such as socio-economic class, diet or genes) can be dealt with only in two ways: by using randomization in a trial design and by using matching in an etiological case-control study. Cases and controls are then matched on a 'proxy' factor, such as being a partner or sibling.

Examples of matching on unknown factors:

1) In a study to assess a relation between tonsillectomy and Hodgkin's disease, a confounding effect was expected of certain (unknown) genes that could lead to both an increased susceptibility for infections (and hence tonsillectomy) and Hodgkin's disease. In an unmatched case-control study, one would stratify in the analysis on the presence of those genes, but, as they were unknown, this was not possible. Therefore, the investigators decided to match on a proxy factor for similar genes, and siblings were taken as controls.
2) The ultimate example of a matched design is the case-crossover study, where cases and controls are the same individuals. Here, only exposure is compared at different time-points, shortly before the event and at a random moment. This way, all fixed characteristics that could confound the association (e.g. age, sex, genes) are controlled for. The case-crossover design is also particularly useful to control for unknown confounders and has been used to study acute triggers of myocardial infarction or the effect of using mobile phones on traffic accidents.

One of the disadvantages of matching is that, while used to control confounding, it actually introduces confounding. Matching in a case-control study seems intuitive, and to fulfill the ‘ceteris paribus’ principle. It is, however, vastly different from matching in a cohort design, and still necessitates a stratified (‘matched’) analysis. In a trial design, individuals are allocated to the compared groups on the basis of chance (randomization), hence ensuring generally that confounding variables are equally distributed over the 2 groups of exposure. This ensures that both groups are equal in every respect, except for the exposure of interest. Subsequently, these factors can be ignored in the analysis. Analogously, in an observational cohort study, in which individuals with and without an exposure of interest are followed over time and rates of disease are compared, the researcher may match those with the exposure of interest on potentially confounding variables to the unexposed. Again, the groups are equal in all factors except the exposure of interest*.* This makes it tempting to believe that the same will happen when matching takes place in a case-control study. However, this is not the case, and the analogy is false. In the trial and cohort designs, cases (individuals with the outcome of interest) will arise in both groups of exposure during the follow-up of the study, while the other participants reach the end of the follow-up remaining non-cases.

In an *unmatched* case-control design, the same cases as those that arose in the trial or cohort are included and the controls are sampled from the non-cases. When this sampling occurs randomly, it ensures that the exposure odds in the controls is that of the original population. So, when in a trial or cohort study incidence rates in each group of exposure (i_1_/N_1_ in exposed and i_0_/N_0_ in unexposed) can be calculated, and their ratio (i_1_/N_1_ / i_0_/N_0_ = i_1_/i_0_ / N_1_/N_0_) will be the relative risk (RR), in a case-control study the cases will yield their exposure odds (i_1_/i_0_) and the controls the exposure odds in the population (n_1_/n_0_), and the odds ratio estimates the relative risk. Note that in a cohort study the cases arise from both groups of exposure, and that one would not at all expect the cases to resemble the non-cases: in fact, they will exhibit a higher frequency of all risk factors than the non-cases. Therefore, while intuitive, it does not follow the 'ceteris paribus' principle to try and make cases resemble controls in a case-control study.

In a *matched* case-control design, the control subjects are not a random sample from the non-cases, as they need to be selected on the matching factors: certain characteristics that are associated with the exposure and the outcome of interest (e.g., same age, sex, life style). This implies that matched controls will inevitably be made more similar with respect to the exposure of interest than random controls would have been. A new confounding structure is introduced, and the strength of the association between exposure and outcome of interest will be underestimated. This problem can be solved by the standard solution to confounding: performing a stratified analysis, for within strata of the confounding factor, the association between exposure and outcome is unconfounded due to the absence of variation of the confounding variable in that stratum. In other words, the solution is to estimate the association of the exposure of interest with the outcome conditional on the confounding variable.

There are two types of matching, both of which are drawing controls from the population in a stratified fashion. In ‘*frequency matching’* a control group is selected in such a way that its distribution of the matching variables becomes similar to that of the cases, without there being 1:1 matching of individual cases and controls. In this case each of the strata, defined by the matching variable, contain a number of cases and controls, and since these numbers will not represent the actual distribution of the confounder, and thus of the associated risk factor of interest in the population, the crude comparison of exposure in cases and controls is no longer fair. This is essentially the same as saying that the strata are weighted in a different way than when an unstratified sample had been taken: confounding has been introduced. Within each stratum, the odds ratio is not affected by the weighting of the strata, and so a stratified analysis removes the confounding. The stratumspecific odds ratios are reweighted into a pooled estimator, such as a Mantel-Henszel odds ratio, or a maximum likelihood regression coefficient in an unconditional logistic regression model. It should be noted that this kind of matching can only be done on co-variates that are known, and therefore is not done to control for confounding, but to increase efficiency in case some strata would be small or unbalanced between cases and controls.

A more "extreme" form is *individual matching*, where for each case a control subject with the relevant characteristics is sought. This can be seen as extreme stratified sampling: each stratum contains only one case and only one control. Again, within each stratum there is no confounding since the confounder (matching variable) is invariant within the stratum, and, although the individual odds ratio is meaningless, the contribution to the odds ratio of each stratum is fair. The solution is again a stratified analysis over all strata of one case-control pair, which can again be done by calculation of a Mantel-Haenzel odds ratio (MH(OR)), or by a maximum likelihood regression coefficient from a conditional logistic regression model.

It can be shown that the MH(OR) is simply the ratio of discordant pairs:

As each stratum consists of 2 individuals, there are only 4 possible outcomes per stratum: 1) both case and control were exposed; 2) the case was exposed and the control was not; 3) the control was exposed and the case was not; 4) both case and control were not exposed:

| Exposure | | | | | | | | | |
| --- | --- | --- | --- | --- | --- | --- | --- | --- | --- |
|  | + | - | + | - | + | - | + | - | Total for each table |
| Case | 1 | 0 | 1 | 0 | 0 | 1 | 0 | 1 | 1 |
| Control | 1 | 0 | 0 | 1 | 1 | 0 | 0 | 1 | 1 |
|  |  |  |  |  |  |  |  |  |  |
| Total | 2 | 0 | 1 | 1 | 1 | 1 | 0 | 2 | 2 |
|  | n_11_ | | n_10_ | | n_01_ | | n_00_ | | n |

In n_ij_, the first subscript (i) represents the exposure status of the case and the second (j) that of the control (0=unexposed, 1= exposed).

The summary of the MH estimate will then be:

[(n_11_ * (1*0)) + (n_10_ * (1*1)) + (n_01_ * (0*0)) + (n_00_ * (0*1))] / 2

[(n_11_ * (0*1)) + (n_10_ * (0*0)) + (n_01_ * (1*1)) + (n_00_ * (1*0))] / 2

=

[(n_11_ * 0) + (n_10_ * 1) + (n_01_ * 0) + (n_00_ * 0)] / 2

[(n_11_ * 0) + (n_10_ * 0) + (n_01_ * 1) + (n_00_ * 0)] / 2

=	n_10_

n_01_

This shows that the concordant pairs do not contribute information to the summary MH estimate, which is the ratio of the numbers of discordant pairs (with McNemar’s test as a statistical test for the null-hypothesis that MH(OR)=1).

When other confounding factors are to be taken into account in an individually matched case-control study that are not taken into account by the matching, stratification is not possible, as this would break up the paired matching. Therefore, conditional logistic regression techniques have to be used. This is a modification of unconditional logistic regression, in which the likelihood has been rewritten to take account of the matching. Parameter estimates are obtained by looking at the values of the explanatory variables among cases and controls within each stratum. Essentially, the odds ratio of the exposure of interest is estimated conditional on the matching variable, while the other co-variates are estimated unconditional on the matching variable. For an unmatched study an unconditional logistic model is used, in which

$$\mathrm{logit}(X_{1},X_{2})=\ln(\frac{P(Y=1|X_{1},X_{2})}{P(Y=0|X_{1},X_{2})})=\beta_{0}+\beta_{1}X_{1}+\beta_{2}X_{2}$$

$Y$: outcome of interest (0/1)

$X_{1}$: exposure(s)

$X_{2}$: confounder(s)

Parameters are estimated via Maximum Likelihood

It can be shown that e^βi^ is the odds ratio for each X_i._ Confounding will occur if $X_{2}$ has an effect ($\beta_{2}\neq0$) and $X_{1}$ and $X_{2}$are correlated.

Conditional regression is obtained by computing the conditional likelihood within a stratum given the total number of cases in a stratum. The general formulas get complicated if there are more cases in a stratum. The simplest is the situation of 1 case and 1 control per stratum. The conditional likelihood per stratum (loosely speaking: the probability that the case is the case) is given by

$$\begin{matrix} L=\frac{P(Y_{case}=1)}{P(Y_{case}=1)+P(Y_{cont}=1)}=\frac{\exp(X_{1,case}\beta_{1}+X_{2,case}\beta_{2})}{\exp(X_{1,case}\beta_{1}+X_{2,case}\beta_{2})+\exp(X_{1,cont}\beta_{1}+X_{2,cont}\beta_{2})} \\ =\frac{\exp((X_{1,case}-X_{1,cont})\beta_{1}+(X_{2,case}-X_{2,cont})\beta_{2})}{1+\exp((X_{1,case}-X_{1,cont})\beta_{1}+(X_{2,case}-X_{2,cont})\beta_{2})} \end{matrix}$$

The total likelihood is the product over all strata.

If there is matching in the strata on the confounder$X_{2}$, the $X_{2}$-term drops from the equation. It is also clear from this expression that pairs with equal exposure do not contribute to the estimation of $\beta_{1}$. In the simple case of a dichotomous exposure and matching on confounders, the Maximum Likelihood estimate of $\beta_{1}$ coincides with the Mantel-Haenszel estimator and is given by $\ln(n_{10}/n_{01})$. (ln(number of pairs in which case is exposed and control not/number of pairs in which control is exposed and case not).

Matching in a case-control study has other disadvantages. An important one is that the effect of the matching factor as a potential risk factor can no longer be examined, since all differences between cases and controls have been taken out. Furthermore, it can be difficult to find suitable matches and there is the possibility of "overmatching". This is something that can occur in two ways: when matching is performed on a variable that is strongly correlated with exposure but not with disease, it will result in a loss of power (a large number of pairs that are not discordant and do not contribute to the risk estimate). The second type of overmatching happens when matching is performed on a variable in the causal pathway between exposure of interest and outcome. This will result in a biased estimate of the risk of exposure. Nevertheless, apart from this particular situation, no validity problem is introduced by matching, as long as the appropriate analytical techniques are used.

In summary: matching intuitively seems a method to control for confounding, but it in fact introduces confounding and necessitates a stratified analysis. The strongest advantage of matching is to allow control for the effect of factors that cannot easily be measured, as this is difficult in any epidemiological design other than a randomized controlled trial. For example, recruiting siblings as controls may help to control for genetic factors, and recruiting neighbours may help to control for socio-economic factors or access to health services.

**A special case of matching: sex-reversed matching**

Matching is often performed on sex and age, as these are frequently considered to be confounding factors. In our study, the Multiple Environmental and Genetic Assessment of risk factors for venous thrombosis (the MEGA study), we did not exactly do so, but took partners as control subjects. By doing this, we approximately matched on age, but our main consideration was to use partnership as a 'proxy' for many factors that are confounders but difficult to control for (such as socioeconomic class), or for factors that are unknown. Another advantage of this approach is that we expected that choosing this control group would ensure the highest response rate, even among those with risk factors such as malignancy, recent surgery, pregnancy and so on. Obviously, the result of this procedure was that for the vast majority of cases 'sex-reversed matching' occurred.

There is a possibility that confounding occurs using the sex-reversed matching method when sex and exposure are related and when both are risk factors for the disease of interest, in other words, if, over the pairs, $X_{2,case}-X_{2,cont}$ is correlated with $X_{1,case}-X_{1,cont}$. This can be solved by adjusting for sex in the analysis*.* However, since stratification is already maximized in the MH method (1 to 1 pairs), this method does not allow for further stratification. In a conditional logistic regression model, the influence of factors other than the matched sets can be assessed as in an unconditional logistic model and therefore sex can simply be added to the model. This way, the effect of the exposure of interest is estimated while taking into account the separate effect of sex.

To illustrate that this procedure did not lead to any form of bias, and that it allowed to control for a possible confounding effect of sex, we simulated all conceivable situations of potential confounding (1-6). From these simulations it can be concluded that, firstly, the results of conditional logistic regression are invariably correct (resulting odds ratio is a good approximation of the underlying risk ratio in the population), except when sex is distributed unevenly over the cases and is itself associated with the disease; and, secondly, that this can be corrected fully when sex is added to the model, again leading to an unconfounded odds ratio.

**General reading:**

Breslow NE, Day NE. Statistical methods in cancer research; Vol 1 – The analysis of case-control studies. Lyon, France: IARC scientific publications; 1980.

Schlesselman JJ. Case-control studies. Design, conduct, analysis. New York, USA: Oxford University Press; 1982.

Rothman KJ. Modern Epidemiology. Boston, USA: Little, Brown and Company;1986.

Clayton D, Hills M. Statistical Models in Epidemiology. New York, USA: Oxford University Press; 1993.

**Situation 1: The exposure is equally common in both sexes; it is more frequent within couples; it is not a risk factor for disease (RR=1) and neither is sex.**

Starting points for simulation:

Population size of 10.000 men and 10.000 women

Exposure (E1) is	not related to sex: present in 10% of men and 10% of women

more frequent in couples (50% of E1+ women find E1+ men)

not a risk factor for disease (E1+ leads to disease in 1%; E1- leads to disease in 1%: RR =1.0)

Sex is not a risk factor for disease

| E1 is present in 10% of women and 10% of men | Number of cases in E1+ (1%) and E1- (1%) | Pair formation: 50% of E1+ women find E1+ men | Number of cases in opposite sex |  |
| --- | --- | --- | --- | --- |
| 1000 We+ | 10 We+C+ | 5 Me+ | 0.05 Me+C+ |  |
|  |  |  | 4.95 Me+C- | a |
|  |  | 5 Me- | 0.05 Me-C+ |  |
|  |  |  | 4.95 Me-C- | b |
|  | 990 We+C- | 495 Me+ | 4.95 Me+C+ | c |
|  |  |  | 490.05 Me+C- |  |
|  |  | 495 Me- | 4.95 Me-C+ | d |
|  |  |  | 490.05 Me-C- |  |
| 9000 We- | 90 We-C+ | 5 Me+ | 0.05 Me+C+ |  |
|  |  |  | 4.95 Me+C- | e |
|  |  | 85 Me- | 0.85 Me-C+ |  |
|  |  |  | 84.15 Me-C- | f |
|  | 8910 We-C- | 495 Me+ | 4.95 Me+C+ | g |
|  |  |  | 490.05 Me+C- |  |
|  |  | 8415 Me- | 84.15 Me-C+ | h |
|  |  |  | 8330.85 Me-C- |  |
|  |  |  |  |  |

*Annotation (for all 6 simulations): W: women; M: men; e+: exposed; e-: not exposed; C+: case; C-: control.*

Taking all cases with their corresponding partner control (rounded to whole persons):

a: 5 We+C+ with 5 Me+C-

b: 5 We+C+ with 5 Me-C-

c: 5 We+C- with 5 Me+C+

d: 5 We+C- with 5 Me-C+

e: 5 We-C+ with 5 Me+C-

f: 84 We-C+ with 84 Me-C-

g: 5We-C- with 5 Me+C+

h: 84 We-C- with 84 Me-C+

This gives the following 2x2 table:

|  |  | Controls | |
| --- | --- | --- | --- |
|  |  | E1+ | E1- |
| Cases | E1+ | 10  (a+c) | 10  (b+g) |
|  | E1- | 10  (d+e) | 168  (f+h) |

**Results from the case-control analysis:**

Matched OR (MH): 10/10= 1.0

Conditional Logistic Regression OR: 1.0

Conditional Logistic Regression OR adjusted for sex: 1.0

**Situation 2: The exposure is more common in men; it is more frequent within couples; it is not a risk factor for disease (RR=1) and neither is sex.**

Starting points for simulation:

Population size of 10.000 men and 10.000 women

Exposure (E2) is	related to sex: present in 20% of men and 10% of women

more frequent in couples (50% of E2+ women find E2+ men)

not a risk factor for thrombosis (E2+ leads to disease in 1%; E2- leads to disease in 1%: RR =1.0)

and sex is not a risk factor for disease

| E2 is present in 10% of women and 20% of men | Number of cases in E2+ (1%) and E2- (1%) | Pair formation: 50% of E2+ women find E2+ men | Number of cases in opposite sex |  |
| --- | --- | --- | --- | --- |
| 1000 We+ | 10 We+C+ | 5 Me+ | 0.05 Me+C+ |  |
|  |  |  | 4.95 Me+C- | a |
|  |  | 5 Me- | 0.05 Me-C+ |  |
|  |  |  | 4.95 Me-C- | b |
|  | 990 We+C- | 495 Me+ | 4.95 Me+C+ | c |
|  |  |  | 490.05 Me+C- |  |
|  |  | 495 Me- | 4.95 Me-C+ | d |
|  |  |  | 490.05 Me-C- |  |
| 9000 We- | 90 We-C+ | 15 Me+ | 0.15 Me+C+ |  |
|  |  |  | 14.85 Me+C- | e |
|  |  | 75 Me- | 0.75 Me-C+ |  |
|  |  |  | 74.25 Me-C- | f |
|  | 8910 We-C- | 1485 Me+ | 14.85 Me+C+ | g |
|  |  |  | 1470.15 Me+C- |  |
|  |  | 7425 Me- | 74.25 Me-C+ | h |
|  |  |  | 7350.75 Me-C- |  |
|  |  |  |  |  |

Taking all cases with their corresponding partner control (rounded to whole persons):

a: 5 We+C+ with 5 Me+C-

b: 5 We+C+ with 5 Me-C-

c: 5 We+C- with 5 Me+C+

d: 5 We+C- with 5 Me-C+

e: 15 We-C+ with 15 Me+C-

f: 74 We-C+ with 74 Me-C-

g: 15 We-C- with 15 Me+C+

h: 74 We-C- with 74 Me-C+

This gives the following 2x2 table:

|  |  | Controls | |
| --- | --- | --- | --- |
|  |  | E2+ | E2- |
| Cases | E2+ | 10  (a+c) | 20  (b+g) |
|  | E2- | 20  (d+e) | 148  (f+h) |

**Results from the case-control analysis:**

Matched OR (MH): 20/20= 1.0

Conditional Logistic Regression OR: 1.0

Conditional Logistic Regression OR adjusted for sex: 1.0

**Situation 3: The exposure is equally common in both sexes; it is more frequent within couples; it is a risk factor for disease (RR =4) but sex is not.**

Starting points for simulation:

Population size of 10.000 men and 10.000 women

Exposure (E3) is	not related to sex: present in 10% of men and 10% of women

more frequent in couples (50% of E3+ women find E3+ men)

a risk factor for thrombosis: E3+ leads to disease in 4%, E3- leads to disease in 1% (RR = 4)

and sex is not a risk factor for disease

| E3 is present in 10% of women and 10% of men | Number of cases in E3+ (4%) and E3- (1%) | Pair formation: 50% of E3+ women find E3+ men | Number of cases in opposite sex |  |
| --- | --- | --- | --- | --- |
| 1000 We+ | 40 We+C+ | 20 Me+ | 0.8 Me+C+ |  |
|  |  |  | 19.2 Me+C- | a |
|  |  | 20 Me- | 0.2 Me-C+ |  |
|  |  |  | 19.8 Me-C- | b |
|  | 960 We+C- | 480 Me+ | 19.2 Me+C+ | c |
|  |  |  | 460.8 Me+C- |  |
|  |  | 480 Me- | 4.8 Me-C+ | d |
|  |  |  | 475.2 Me-C- |  |
| 9000 We- | 90 We-C+ | 5 Me+ | 0.2 Me+C+ |  |
|  |  |  | 4.8 Me+C- | e |
|  |  | 85 Me- | 0.85 Me-C+ |  |
|  |  |  | 84.15 Me-C- | f |
|  | 8910 We-C- | 495 Me+ | 19.8 Me+C+ | g |
|  |  |  | 475.2 Me+C- |  |
|  |  | 8415 Me- | 84.2 Me-C+ | h |
|  |  |  | 8330.8 Me-C- |  |
|  |  |  |  |  |

Taking all cases with their corresponding partner control (rounded to whole persons):

a: 19 We+C+ with 19 Me+C-

b: 20 We+C+ with 20 Me-C-

c: 19 We+C- with 19 Me+C+

d: 5 We+C- with 5 Me-C+

e: 5 We-C+ with 5 Me+C-

f: 84 We-C+ with 84 Me-C-

g: 20 We-C- with 20 Me+C+

h: 84 We-C- with 84 Me-C+

This gives the following 2x2 table:

|  |  | Controls | |
| --- | --- | --- | --- |
|  |  | E3+ | E3- |
| Cases | E3+ | 38  (a+c) | 40  (b+g) |
|  | E3- | 10  (d+e) | 168  (f+h) |

**Results from the case-control analysis:**

Matched OR (MH): 40/10= 4.0

Conditional Logistic Regression OR: 4.0

Conditional Logistic Regression OR adjusted for sex: 4.0

**Situation 4: The exposure is more common in men; it is more frequent within couples; it is a risk factor for disease (RR =4) but sex is not.**

Starting points for simulation:

Population size of 10.000 men and 10.000 women

Exposure (E4) is	related to sex: present in 20% of men and 10% of women

more frequent in couples (50% of E4+ women find E4+ men)

a risk factor for thrombosis: E4+ leads to disease in 4%, E4- leads to disease in 1% (RR = 4)

and sex is not a risk factor for disease

| E4 is present in 10% of women and 20% of men | Number of cases in E4+ (4%) and E4- (1%) | Pair formation: 50% of E4+ women find E4+ men | Number of cases in opposite sex |  |
| --- | --- | --- | --- | --- |
| 1000 We+ | 40 We+C+ | 20 Me+ | 0.8 Me+C+ |  |
|  |  |  | 19.2 Me+C- | a |
|  |  | 20 Me- | 0.2 Me-C+ |  |
|  |  |  | 19.8 Me-C- | b |
|  | 960 We+C- | 480 Me+ | 19.2 Me+C+ | c |
|  |  |  | 460.8 Me+C- |  |
|  |  | 480 Me- | 4.8 Me-C+ | d |
|  |  |  | 475.2 Me-C- |  |
| 9000 We- | 90 We-C+ | 15 Me+ | 0.6 Me+C+ |  |
|  |  |  | 14.4 Me+C- | e |
|  |  | 75 Me- | 0.75 Me-C+ |  |
|  |  |  | 74.25 Me-C- | f |
|  | 8910 We-C- | 1485 Me+ | 59.4 Me+C+ | g |
|  |  |  | 1425.6 Me+C- |  |
|  |  | 7425 Me- | 74.25 Me-C+ | h |
|  |  |  | 7350.75 Me-C- |  |
|  |  |  |  |  |

Taking all cases with their corresponding partner control (rounded to whole persons):

a: 19 We+C+ with 19 Me+C-

b: 20 We+C+ with 20 Me-C-

c: 19 We+C- with 19 Me+C+

d: 5 We+C- with 5 Me-C+

e: 14 We-C+ with 14 Me+C-

f: 74 We-C+ with 74 Me-C-

g: 59 We-C- with 59 Me+C+

h: 74 We-C- with 74 Me-C+

This gives the following 2x2 table:

|  |  | Controls | |
| --- | --- | --- | --- |
|  |  | E4+ | E4- |
| Cases | E4+ | 38  (a+c) | 79  (b+g) |
|  | E4- | 19  (d+e) | 148  (f+h) |

**Results from the case-control analysis:**

Matched OR (MH): 79/19= 4.16

Conditional Logistic Regression OR: 4.16

Conditional Logistic Regression OR adjusted for sex: 4.06

**Situation 5: The exposure is more common in men; it is more frequent within couples; it is not a risk factor for disease (RR =1) but sex is.**

Starting points for simulation:

Population size of 10.000 men and 10.000 women

Exposure (E5) is	related to sex: present in 20% of men and 10% of women

more frequent in couples (50% of E5+ women find E5+ men)

not a risk factor for thrombosis (RR =1))

and sex is a risk factor for disease: 3% of men get the disease, 1% of women

| E5 is present in 10% of women and 20% of men | Number of cases in E5+ (1%) and E5- (1%) | Pair formation: 50% of E5+ women find E5+ men | Number of cases in opposite sex |  |
| --- | --- | --- | --- | --- |
| 1000 We+ | 10 We+C+ | 5 Me+ | 0.15 Me+C+ |  |
|  |  |  | 4.85 Me+C- | a |
|  |  | 5 Me- | 0.15 Me-C+ |  |
|  |  |  | 4.85 Me-C- | b |
|  | 990 We+C- | 495 Me+ | 14.85 Me+C+ | c |
|  |  |  | 480.15 Me+C- |  |
|  |  | 495 Me- | 14.85 Me-C+ | d |
|  |  |  | 480.15 Me-C- |  |
| 9000 We- | 90 We-C+ | 15 Me+ | 0.45 Me+C+ |  |
|  |  |  | 14.55 Me+C- | e |
|  |  | 75 Me- | 2.25 Me-C+ |  |
|  |  |  | 72.75 Me-C- | f |
|  | 8910 We-C- | 1485 Me+ | 44.55 Me+C+ | g |
|  |  |  | 1440.45 Me+C- |  |
|  |  | 7425 Me- | 222.75 Me-C+ | h |
|  |  |  | 7202.25 Me-C- |  |
|  |  |  |  |  |

Taking all cases with their corresponding partner control (rounded to whole persons):

a: 5 We+C+ with 5 Me+C-

b: 5 We+C+ with 5 Me-C-

c: 15 We+C- with 15 Me+C+

d: 15 We+C- with 15 Me-C+

e: 15 We-C+ with 15 Me+C-

f: 73 We-C+ with 73 Me-C-

g: 45 We-C- with 45 Me+C+

h: 223 We-C- with 223 Me-C+

This gives the following 2x2 table:

|  |  | Controls | |
| --- | --- | --- | --- |
|  |  | E5+ | E5- |
| Cases | E5+ | 20 | 50 |
|  | E5- | 30 | 296 |

**Results from the case-control analysis:**

Matched OR (MH): 50/30= 1.67

Conditional Logistic Regression OR: 1.67

Conditional Logistic Regression OR adjusted for sex: 1.0

**Situation 6: The exposure is more common in men; it is more frequent within couples; it is a risk factor for disease (RR =4) and sex is too.**

Starting points for simulation:

Population size of 10.000 men and 10.000 women

Exposure (E6) is	related to sex: present in 20% of men and 10% of women

more frequent in couples (50% of E6+ women find E6+ men)

a risk factor for thrombosis: E6+ leads to disease in 4%, E6- leads to disease in 1% (RR = 4)

and sex is a risk factor for disease: 3% of men get the disease, 1% of women:

assume multiplicativity: of E6- men 3% get the disease, E6+ men 12% get the disease, of E6- women 1% get the disease, of E6+ women 4% get the disease.

| E6 is present in 10% of women and 20% of men | Number of cases in E6+ (4%) and E6- (1%) women | Pair formation: 50% of E6+ women find E6+ men | Number of cases in men: E6+ (12%) and E6- (3%) |  |
| --- | --- | --- | --- | --- |
| 1000 We+ | 40 We+C+ | 20 Me+ | 2.4 Me+C+ |  |
|  |  |  | 17.6 Me+C- | a |
|  |  | 20 Me- | 0.6 Me-C+ |  |
|  |  |  | 19.4 Me-C- | b |
|  | 960 We+C- | 480 Me+ | 57.6 Me+C+ | c |
|  |  |  | 422.4 Me+C- |  |
|  |  | 480 Me- | 14.4 Me-C+ | d |
|  |  |  | 465.6 Me-C- |  |
| 9000 We- | 90 We-C+ | 15 Me+ | 1.8 Me+C+ |  |
|  |  |  | 13.2 Me+C- | e |
|  |  | 75 Me- | 2.25 Me-C+ |  |
|  |  |  | 72.75 Me-C- | f |
|  | 8910 We-C- | 1485 Me+ | 178.2 Me+C+ | g |
|  |  |  | 1306.8 Me+C- |  |
|  |  | 7425 Me- | 222.75 Me-C+ | h |
|  |  |  | 7202.25 Me-C- |  |
|  |  |  |  |  |

Taking all cases with their corresponding partner control (rounded to whole persons):

a: 18 We+C+ with 18 Me+C-

b: 19 We+C+ with 19 Me-C-

c: 58 We+C- with 58 Me+C+

d: 14 We+C- with 14 Me-C+

e: 13 We-C+ with 13 Me+C-

f: 73 We-C+ with 73 Me-C-

g: 178 We-C- with 178 Me+C+

h: 223 We-C- with 223 Me-C+

This gives the following 2x2 table:

|  |  | Controls | |
| --- | --- | --- | --- |
|  |  | E6+ | E6- |
| Cases | E6+ | 76  (a+c) | 197  (b+g) |
|  | E6- | 27  (d+e) | 296  (f+h) |

**Results from the case-control analysis:**

Matched OR (MH): 197/27= 7.3

Conditional Logistic Regression OR: 7.3

Conditional Logistic Regression OR adjusted for sex: 4.2
